# Unraveling the influence of microbial necromass on subsurface microbiomes: metabolite utilization and community dynamics

**DOI:** 10.1093/ismeco/ycaf006

**Published:** 2025-01-29

**Authors:** Brianna K Finley, Brandon C Enalls, Markus de Raad, Mariam Al Said, Mingfei Chen, Dominique C Joyner, Terry C Hazen, Trent R Northen, Romy Chakraborty

**Affiliations:** Department of Ecology, Earth and Environmental Sciences Area, Lawrence Berkeley National Laboratory, Berkeley, CA 94720, United States; Department of Ecology, Earth and Environmental Sciences Area, Lawrence Berkeley National Laboratory, Berkeley, CA 94720, United States; Environmental Genomics and Systems Biology Division, Lawrence Berkeley National Laboratory, Berkeley, CA 94720, United States; Department of Ecology, Earth and Environmental Sciences Area, Lawrence Berkeley National Laboratory, Berkeley, CA 94720, United States; Department of Ecology, Earth and Environmental Sciences Area, Lawrence Berkeley National Laboratory, Berkeley, CA 94720, United States; Department of Civil and Environmental Engineering, University of Tennessee, Knoxville, Tennessee 37996, United States; Department of Civil and Environmental Engineering, University of Tennessee, Knoxville, Tennessee 37996, United States; Genomics Sciences Division, Oak Ridge National Laboratory, Oak Ridge, Tennessee 37830, United States; Environmental Genomics and Systems Biology Division, Lawrence Berkeley National Laboratory, Berkeley, CA 94720, United States; Joint Genome Institute, Lawrence Berkeley National Laboratory, Berkeley, CA 94720, United States; Department of Ecology, Earth and Environmental Sciences Area, Lawrence Berkeley National Laboratory, Berkeley, CA 94720, United States

**Keywords:** necromass, metabolites, subsurface, microbial enrichments, organic carbon, turnover

## Abstract

The role of microbial necromass (nonliving microbial biomass), a significant component of belowground organic carbon, in nutrient cycling and its impact on the dynamics of microbial communities in subsurface systems remains poorly understood. It is currently unclear whether necromass metabolites from various microbes are different, whether certain groups of metabolites are preferentially utilized over others, or whether different microbial species respond to various necromass metabolites. In this study, we aimed to fill these knowledge gaps by designing enrichments with necromass as the sole nutrient source for subsurface microbial communities. We used the soluble fraction of necromass from bacterial isolates belonging to *Arthrobacter*, *Agrobacterium*, and *Pseudomonas* genera, and our results indicate that metabolite composition of necromass varied slightly across different strains but generally included amino acids, organic acids, and nucleic acid constituents. *Arthrobacter*-derived necromass appeared more recalcitrant. Necromass metabolites enriched diverse microbial genera, particularly *Massilia* sp. responded quickly regardless of the necromass source. Despite differences in necromass utilization, microbial community composition converged rapidly over time across the three different necromass amendments. Uracil, xanthine, valine, and phosphate-containing isomers were generally depleted over time, indicating microbial assimilation for maintenance and growth. However, numerous easily assimilable metabolites were not significantly depleted, suggesting efficient necromass recycling and the potential for necromass stabilization in systems. This study highlights the dynamic interactions between microbial necromass metabolites and subsurface microbial communities, revealing both selective utilization and rapid community and necromass convergence regardless of the necromass source.

## Introduction

Understanding the fate and dynamics of natural organic matter (OM) transformations in subsurface ecosystems is crucial for comprehending their role in global carbon and nutrient cycling. Part of the challenge is that natural OM composition is highly heterogeneous, and can vary widely based on biotic, abiotic, and spatiotemporal factors across ecosystems [1, 2]. Additionally, different components in OM can alter microbial community composition and metabolic responses in these different ecosystems [3–7]. Up to 80% of belowground OM is necromass, the remnants of microbial activity and cellular decay [8]. This source of OM is increasingly recognized as an important contributor toward OM formation and persistence and microbial-mediated turnover in soil [8–11], marine sediments [12], and terrestrial sediments and groundwater [5, 13–15].

The role of necromass on persistent OM has gained increasing attention, yet characterization of the constituents, as well as measurement of the relative contribution of necromass to persistent OM is challenging. In the context of OM persistence, necromass cycling can be understood in four broad yet dynamic processes: production, recycling, stabilization, and destabilization, as detailed in Buckeridge *et al*. (2022) [9]. Some of the necromass produced is retained and recycled in ecosystems as new living biomass or lost via respiration [9]. Some other constituents are protected by stabilization or re-released by destabilization as a result of biotic and abiotic transformations based on necromass chemistry, the decomposer microbial community, its spatial location, and organomineral associations [9]. The efficiency by which microorganisms interact with necromass may affect its stabilization, and therefore persistence in belowground OM.

To date, studies investigating necromass as a component of belowground OM have focused largely in the context of surface soils (i.e. the top 10 cm) [16], yet necromass is critically important as a carbon and nutrient source in subsurface environments. Terrestrial shallow subsurface and groundwater communities rarely receive direct plant carbon inputs [14–17] and tend to be OM and carbon limited. Furthermore, the carbon present in the subsurface displays high mean residence times and is enriched in microbial-derived compounds [18]. Dissolved OM (DOM) from sediment is one of the primary forms of carbon and nutrients available for microbial sustenance in the subsurface [14, 19, 20], and microbial necromass that is generally rich in carbohydrates, amino acids, organic acids, fatty acids, sterols and nucleosides [21], is a key contributor to sediment-derived DOM [22]. Previous research showed that carbon amendments in the form of sediment-derived DOM and lab-generated microbial necromass led to growth of more diverse microbial communities than simple single compounds such as glucose, acetate or benzoate [5], demonstrating that subsurface microbial communities are more adapted to utilizing naturally-occurring OM than single carbon sources.

Some compounds in necromass such as extracellular deoxyribonucleic acid (DNA) or amino sugars have been used as a common proxy for quantification of total microbial necromass carbon in soils [17, 23], since amino sugars are enriched in necromass compared to living microbial biomass. Furthermore, peptidoglycan (comprising the amino sugars N-acetyl glucosamine and muramic acid) has been used to estimate contributions of necromass from gram-positive or gram-negative prokaryotes, since it occurs up to 90% of dry weight in Gram-positive cell walls but only 5%–20% of Gram-negative cell walls [24]. However, these proxies are far from perfect. Amino sugars can only be used to trace and quantify the fate of cell wall components from necromass. Extracellular DNA from dead microorganisms has been used both as a necromass amendment and proxy for all necromass from microbes in past studies [25, 26], which has the added benefit of identifying taxa that contributed to necromass.

However, extracellular DNA decomposition may not reflect decomposition rates of other classes of compounds in necromass. Other studies have used various isotope tracer methods to generate and then quantify decomposition rates of necromass [10, 13, 27, 28]. Despite these insights, a critical knowledge gap persists in our understanding of the different metabolites from necromass available for microbial utilization, different microbial processing rates of those metabolites, and eventually the contributions of necromass metabolites to total carbon pools [9, 29, 30]. Recently, a study suggested almost 25% of necromass metabolites were stabilized as OM [28], and other study further linked hydrophilic necromass metabolites to formation of persistent soil OM [31], highlighting the critical role necromass-derived metabolites play in carbon cycling.

The overarching aims for this study were to address these knowledge gaps by elucidating the response of a subsurface microbial community to the soluble fraction of necromass generated from representative subsurface microbial strains to determine (i) whether metabolites from necromass from different bacterial strains differentially affect microbial community development (ii) which metabolites within necromass are readily utilized by microbes, and (iii) if utilization patterns vary across different types of necromass. We generated DOM-necromass from commonly-occurring gram-positive and gram-negative bacterial strains of distinct phylogenetic lineages and used the resulting necromass as the sole carbon source to feed subsurface microbial communities. We used 16S ribosomal ribonucleic acid (rRNA) gene amplicon sequencing to elucidate microbial community response, and metabolomics to characterize the composition of low molecular weight compounds of the initial necromass as well as the dynamic necromass utilization over time. Lastly, we used PICRUSt2 to link specific necromass-responding taxa with potential utilization of necromass metabolites.

## Materials and methods

### Study system

Subsurface sediment was obtained from Area 3 of the Y-12 National Security Complex, at Oak Ridge Reservation (ORR), Oak Ridge, TN, United States (35.97716498 N, −84.27327938 W). The sediment core was collected in March 2023 0.76–1.5 m below ground surface from the vadose zone from a borehole adjoining well M6 within the ENIGMA Subsurface Observatory Network. Sediments were stored at –80°C for one week before reacclimating at 4°C and subsequent extraction of living microbial cells to use as inoculum.

### Necromass generation

To generate necromass to use as an enrichment substrate, three bacterial isolates (gram-negative *Pseudomonas helmanticensis* strain 28C6 and *Agrobacterium tumefaciens* strain RD_MOLAP_06, and gram-positive *Arthrobacter bambusae* strain THG-GM18) were aerobically cultured. These strains were selected because they were previously isolated from subsurface communities within ORR and generally are commonly abundant microbes in different environments. The isolates were cultured separately in a basal medium with 10 mM glucose. The basal medium used comprised 50 ml of a trace elements solution (5 g L^−1^ of K_2_HPO_4_, 2.5 g L^−1^ of MgSO_4_ 7H_2_O, 2.5 g L^−1^ NaCl, 0.05 g L^−1^ MnSO_4_ 4H_2_O, and 0.05 g L^−1^ FeSO_4_ 7H_2_O) and 930 ml DI water adjusted pH to 7.5. After autoclaving, we added 10 ml each of filter-sterilized 100X mineral and mixed vitamins to complete the media [14].

The mineral mixture (pH 6.0) comprised 1.5 g L^−1^ NTA disodium salt, 3 g L^−1^ MgSO_4_ 7H_2_O, 0.5 g L^−1^ MnSO_4_ H_2_O, 1 g L^−1^ NaCl, 0.1 g L^−1^ FeSO_4_ 7H_2_O, 0.1 g L^−1^ CaCl_2_ 2H_2_O, 0.1 g L^−1^ CoCl_2_ 6H_2_O, 0.13 g L^−1^ ZnCl, 0.01 g L^−1^ CuSO_4_ 5H_2_O, 0.01 g L^−1^ AlK(SO_4_)2 12H_2_O, 0.01 g L^−1^ AlK(SO_4_)2 12H_2_O, 0.01 g L^−1^ boric acid, 0.025 g L^−1^ Na_2_MoO_4_ 2H2O, 0.024 g L^−1^ NiCl_2_ 6H_2_O, 0.025 g L^−1^ Na_2_WO_4_ 2H_2_O, and 0.02 g L^−1^ Na_2_SeO_4_. The vitamin mixture comprised 2 mg L^−1^ of d-biotin, 2 mg L^−1^ folic acid 10 mg L^−1^ pyridoxine HCl, 5 mg L^−1^ riboflavin, 5 mg L^−1^ thiamine, 5 mg L^−1^ nicotinic acid, 5 mg L^−1^ pantothenic acid, 0.1 mg L^−1^ vitamin B12, 5 mg L^−1^ p-amino benzoic acid, and 5 mg L^−1^ alpha-lipoic acid.

The isolates were regrown from glycerol stocks in Reasoner’s 2A agar (R2A) plates prior to scaling to 10 ml basal medium amended with 10 mM glucose. Culture purity was verified via microscopy, and when OD_600_ was at least 0.3, cultures were further scaled up to 100 ml fresh basal media with 10 mM glucose. Once cultures were at least 0.3 OD_600_, we aliquoted into several 50 ml Falcon tubes and centrifuged for 10 min at 10 000 *g* and the supernatant was discarded. The cell pellets were frozen at −80°C until lysis. Thawed pellets were resuspended in 1 ml of 30 mM sodium bicarbonate buffer each and transferred in 1.5 ml aliquots into “Lysis Matrix A” (MP Bio) bead beating tubes and vortexed at maximum power for 20 min. After vortexing, the bead tubes were centrifuged at 10 000 *g* for 10 min and supernatants were recombined in 15 ml Falcon tubes, filtered through 0.22 μm syringe filters, and stored at -80°C until the incubation’s onset. The filtered lysates from the three strains were verified to not retain living cells by viewing no intact cells via microscopy, as well as plating on R2A medium and observing no bacterial growth, prior to storage at −80°C. Total organic C (TOC) of the lysate was quantified using a Lotix Combustion TOC analyzer (Teledyne, Thousand Oaks, California, United States). Since we used the supernatant from lysed cell preps, this primarily consisted of fully or mostly water soluble necromass constituents such as cytoplasmic proteins, soluble components of RNA and DNA, primary and secondary metabolites and soluble lipids.

### Sediment community incubation with necromass

The inoculum for enrichments was first generated by lightly sonicating 1 g aliquots of homogenized sediment in 9 ml 30 mM sodium pyrophosphate buffer with a 3.8 mm diameter probe and 150 V/T ultrasonic homogenizer (BioLogics, Inc. Manassas, Virginia, United States) at 30 Watts for 30 s five times to release intact cells. The sediment-buffer mixture was then centrifuged at 6500 g for 8 min to pellet the sediment particles, the supernatant with microbial cells was used as inoculum. The subsequent starting concentration of live bacterial cells in each enrichment treatment was on average 3.5 × 10^5^ (±1.0 × 10^5^) cells ml^−1^. Cell count was quantified via flow cytometry using green-fluorescent SYTO 9 dye and red fluorescent propidium iodide (Invitrogen, Thermo Fisher, Waltham, MA, United States).

Each enrichment treatment consisted of 1 ml aliquots of the homogenized inoculum, 30 ppm necromass-carbon (except for the non-necromass control), and enough synthetic groundwater medium [14] to bring the final sample volume to 20 ml. There were four different necromass treatments: 30 ppm C of the three lysed strains (*Arthrobacter*, *Pseudomonas*, and *Agrobacterium spp*.), and a mixed-necromass treatment with 10 ppm C of each lysate strain was added for a total of 30 ppm necromass-carbon. The control lacked necromass amendment. TOC and microbial biomass in these sediments is generally very low (1–5 ppm) [14].

Each replicate was contained in 60 ml glass serum bottles, sealed to prevent contamination, with 40 ml of headspace to allow for aerobic conditions. Samples were incubated at 20°C to mimic lower temperatures in subsurface under aerobic conditions for 14 days to prevent confounding results from further microbial turnover contributing to necromass. Each treatment (in triplicate) was subsampled at the start of the incubation (time 0), and at four subsequent collections: Days 2, 4, 6, and 14 of the experiment. At each collection, duplicate 1 ml aliquots were destructively sampled from each replicate, centrifuged at 10 000 g for 10 min, and the resulting pellet frozen at -80°C for DNA extraction and 16S rRNA gene amplicon sequencing. The supernatants from these samples were measured for pH, then 0.2 μm filtered and frozen at -80°C for downstream metabolomics analyses. Additional 1 ml aliquots were collected for glycerol stocks of the enriched communities, added to 1:1 synthetic groundwater:glycerol, and frozen at −80°C. We measured cell counts from the glycerol stocks using an Attune Nxt acoustic focusing cytometer (Invitrogen, Thermo Fisher) and 100x SYBR Green fluorescent dye.

### LC–MS metabolomics

The supernatant samples from the different timepoints were frozen at −80°C and then freeze-dried (Labconco Freeze-Zone). The dried material was resuspended in 375 μl of LC–MS grade methanol containing internal standards (Table S1). In addition to the samples, there were also extraction controls which consisted of the synthetic groundwater media used during the incubation, without the added necromass or inoculum (Table S2). The solution was vortexed 10 s twice, sonicated in ice water for 15 min, and centrifuged (10 000 *g* for 5 min at 4°C) to pellet insoluble material, and then supernatants were filtered using 0.22-μm polyvinylidene difluoride microcentrifuge filtration devices (Pall) (10 000 *g* for 5 min at 4°C). Metabolites were separated using hydrophilic interaction liquid chromatography (HILIC) for polar metabolomics. Analyses were performed using an InfinityLab Poroshell 120 HILIC-Z column (Agilent, Santa Clare, CA, United States) on an Agilent 1290 stack connected to a Q-Exactive Mass Spectrometer (Thermo Fisher Scientific, Waltham, MA, United States) using ElectroSpray Ionization (ESI). LC–MS/MS and ESI parameters are in Table S1.

### Deoxyribonucleic acid extraction and sequencing

DNA was extracted from pellets using the Qiagen DNeasy PowerLyzer PowerSoil Kit using the manufacturer’s suggested protocol with negative control samples generated during each round of extractions. DNA samples underwent quality control and subsequently sequenced at Novogene Corporation, Inc. with the Illumina MiSeq. Bacterial community composition was analyzed by targeting the V4 region of the 16S gene with 515F (5’-GTGCCAGCMGCCGCGGTAA-3′) and 806R (5′-GGACTACHVGGGTWTCTAAT-3′) primer pairs to generate 300 bp paired-end reads.

### Data analysis

16S rRNA gene amplicon sequences were processed via the Qiime2 dada2 denoised paired-end read pipeline version 2023.9 [32], clustered into ASVs based on a 100%-similarity threshold, assigned taxonomies with Silva version 138 [33], and visualized using the R package phyloseq [34]. ASVs considered contaminants were removed from downstream analysis using a combined frequency and prevalence in negative control sequences (0.1 threshold) [35]. The ASVs of the three necromass strains were removed from the sequencing data for community analyses.

A permutational multivariate ANOVA (PERMANOVA) model was used on a Bray-Curtis community distance matrix with 1000 permutations (vegan::adonis2) in order to assess the effect of necromass treatment and time on community composition. In order to identify potential positively responding taxa to the added necromass, we also performed an Analysis of Compositions of Microbiomes with Bias Correction (ANCOM-BC; [36]) for a differential absolute abundance analysis on log-fold change from the initial community for each sampling period within each necromass treatment. Differential abundance analysis was calculated on a phyloseq object (count data) at the ASV level, Holm-adjusted *P*-values (ɑ = 0.05), 1000 iterations, with taxa considered as structural zeros taken into consideration.

Targeted metabolomic data analysis was performed by comparing sample peaks to a library of analytical standards analyzed under the same conditions. Three parameters were compared: matching m/z, retention time, and fragmentation spectra using Metabolite Atlas [37,38]. We considered metabolites as derived from necromass if they had a minimal peak height of: at least 10 000, significantly greater than the extraction background control, and significantly different from the buffer control (Supplemental Tables S1 and S2). Log2 fold change and associated t-tests (ɑ = 0.05) was calculated from each time point from time-0 for each necromass treatment to determine if a given necromass metabolite was significantly enriched or depleted over time. Heatmaps were visualized with ComplexHeatmap::pheatmap [39].

The functional potential from different necromass-amended microbial communities was imputed by Phylogenetic Investigation of Communities by Reconstruction of Unobserved States (PICRUSt2) [40]. For the PICRUSt2 analysis, we selected ASVs determined to positively respond to the added necromass from the differential abundance ANCOM-BC analysis described previously. ASVs were normalized by predicted 16S rRNA gene copy number abundances and predicted microbial metagenomes using a script provided by PICRUSt2 (v2.5.2) [40]. To correlate metabolic results annotated from PICRUSt2 pipeline with targeted metabolomic data, we used the MetaCyc database [41] to select EC metabolic pathways annotated from PICRUSt2 of the significantly depleted metabolites. Then, we selected the most dominant ASV for each of the 26 genera that significantly increased under added necromass compared to non-necromass treatments from the ANCOM-BC analysis. We additionally conducted growth curves of isolates matching the ASVs of positively-responding taxa (as identified via the ANCOM-BC analysis) in the same incubation conditions using the *Arthrobacter* and *Pseudomonas* necromass to assess independent growth as opposed to community-dependent growth (Supplemental methods, Supplemental Fig. S6). Lastly, we imported necromass-associated metabolites with predictive functional pathways via MetaboAnalyst v6.0 pathway and network-functional analyses [42], to identify potential associations between necromass-metabolites (log2-fold change of Day-14 from time-0) with KEGG orthology (KO) pathways. Overall putative KO abundances were averaged across each treatment, and KOs with NSTI values >0.15 were removed from downstream analysis.

## Results

### Necromass lysate as a carbon substrate for subsurface microbes

Necromass generated from three individual bacterial strains, Gram-positive *Arthrobacter bambusae* strain THG-GM18, and the Gram-negative strains *A. tumefaciens* RD_MOLAP_06 and *Pseudomonas helmanticensis* 28C6, was used to enrich bacterial taxa from a subsurface sediment community. In total, 563.8 ppm of TOC was generated from the *Pseudomonas* strain, 996.5 ppm from *Agrobacterium* and 595 ppm from *Arthrobacter*. In our enrichments, we normalized and added low concentrations of necromass (30 ppm carbon of each necromass) to match the carbon concentration expected in similar subsurface environments [43]. Throughout the incubation, pH levels remained relatively constant across treatments (mean pH 6.5 ± 0.03; Table S3), indicating growth conditions remained optimal and consistent across treatments.

**Figure 1:**
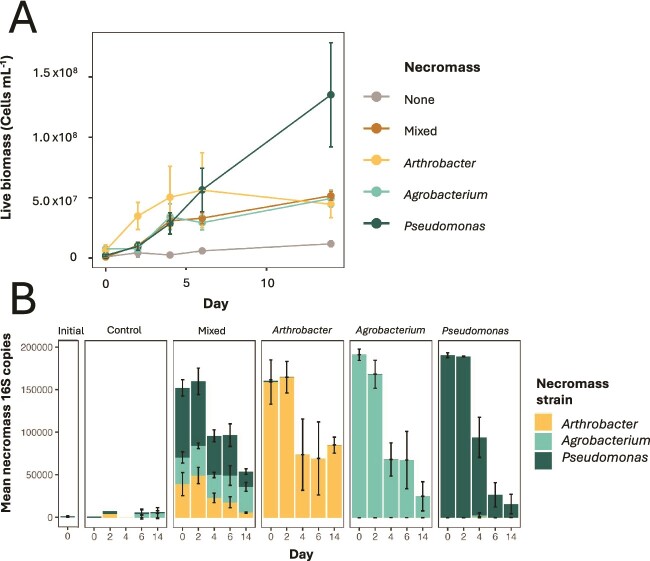
Indications of bacterial utilization of added necromass over the course of the 14-day incubation. (A) Bacterial biomass (cells ml-1), as quantified via flow cytometry at each sampling period (Days 0, 2, 4, 6, and 14). Points and error bars indicate the mean and standard error of the mean (*n* = 3), respectively. (B) Abundance of 16S reads (16S copy number) of the ASVs related to the strains used for necromass generation. Values are mean necromass-strain 16S copies with error bars denoting the standard error of the mean (*n* = 3).

We observed significant growth on all necromass additions. Throughout the 14-day incubation, the control which received no additional necromass besides what was in the inoculum, grew from 9.7 × 10^5^ to 1.2 × 10^7^ cells ml^−1^. Across the necromass treatments, bacterial biomass increased significantly more than the no-necromass control (Fig. 1A). The community from the *Arthrobacter* necromass addition appeared to grow quicker during the early stage of the incubation (Day 2), then stabilized by Day 4. The *Agrobacterium*, *Arthrobacter*, and mixed-necromass treatments all elicited similar bacterial population sizes by the end of the 14-day incubation, while the *Pseudomonas* necromass elicited the most growth by Day 14 (Fig. 1A).

### Necromass utilization as indicated by necromass-amplicon sequence variants

Our results indicate enrichment of diverse subsurface microbial communities on necromass. There was a significant interaction between the source of necromass added and time of incubation on necromass abundance and bacterial growth (Fig. 1). Furthermore, we found evidence of a delayed response in the community’s utilization of microbial necromass. The relative abundance of necromass 16S copies did not significantly decrease regardless of necromass treatment until Day 2. However, there was a rapid decrease in necromass ASV copies between Days 2 and 4 (Fig. 1B ANOVA, *P* < .001; Fig. S1), which continued through the end of the 14-day incubation. *Arthrobacter* necromass also displayed a slower decrease in relative abundance toward the end of the incubation (between Days 6 and 14) compared to the *Pseudomonas* and *Agrobacterium* necromass (ANOVA, *P* < .01). However, when all three necromass strains were mixed, *Arthrobacter* necromass appeared to decrease more quickly compared to when incubated alone (Fig. 1B).

### Necromass metabolite composition and utilization during incubation

**Figure 2:**
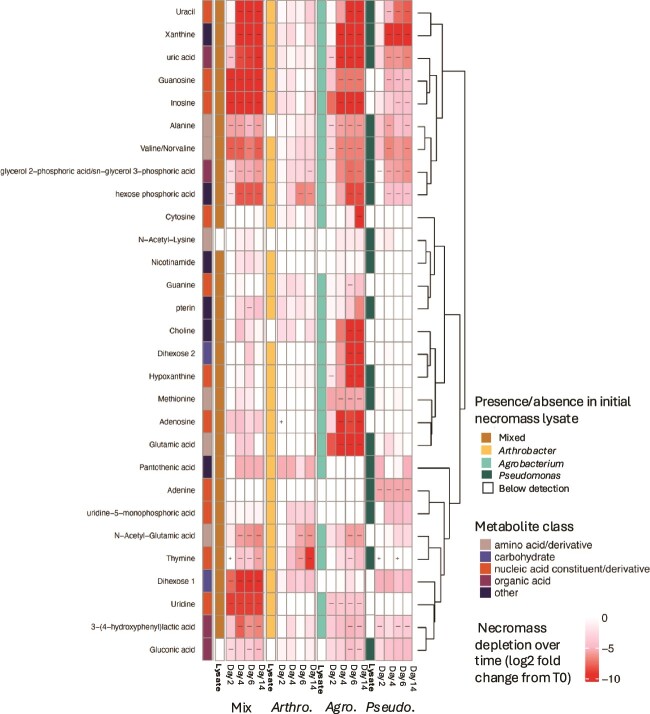
Necromass metabolites initial composition and change over time. The first column of the heatmap indicates the major class of compound of each metabolite. Heatmap shows presence or absence of given metabolite in initial necromass (white cells indicate absence). Metabolite peak height (log scale) across the different necromass treatments and time. The “-” symbols indicate that metabolite was significantly depleted compared to T0 peak height for each treatment (log fold change in peak height from T0 at the four sampling periods: Days 2, 4, 6, and 14 after addition of necromass substrate, α = 0.05, 1000 iterations).

From the targeted LC–MS analysis, 61 metabolites were identified. However, due to the low concentration of necromass-carbon in the samples, numerous metabolites were removed because their origin could not be confidently attributed to the necromass and not the background media. We subsequently identified 29 metabolites with a high confidence of being derived from the various necromass strains (Table 1). The necromass metabolites identified included amino acids and amino acid derivatives, sugars and sugar derivatives, organic acids, nucleobases, nucleosides, nucleotides, and nucleotide derivatives. Twelve of these metabolites were significantly enriched in all three, which included amino acids (valine/norvaline, methionine and glutamic acid), nucleobases (uracil, hypoxanthine, thymine, and xanthine), organic acids (uric acid and the isomers glycerol 2-phosphoric acids/sn-glycerol 3-phosphoric acid), a hexose phosphoric acid, pterin, and pantothenic acid (Table 1; Fig. 2). *Arthrobacter* and *Agrobacterium* necromass contained different dihexoses, whereas *Pseudomonas* necromass did not contain any dihexoses. *Pseudomonas* also had relatively low numbers of nucleosides compared to the other lysates, although it did possess nucleobases.

**Table 1:** Metabolites determined to be present in the various necromass from LC–MS data, sorted by which necromass treatments they were detected in. Other information included are the main class of compounds the metabolite belongs to, the polarity the metabolite was detected in, as well as if the compound is N and/or P containing.

**Detection in necromass**	**Metabolite**	**Class**	**Polarity**	**Nitrogen, phosphorous containing**
Detected in all necromass	Glutamic acid	Amino acid	Positive	N containing
	Methionine	Amino acid	Negative	N containing
	Valine/norvaline	Amino acid	Negative	N containing
	Pantothenic acid	B vitamin	Positive	N containing
	Hypoxanthine	Nucleobase	Positive	N containing
	Thymine	Nucleobase	Negative	N containing
	Uracil	Nucleobase	Negative	N containing
	Glycerol 2-phosphoric acid/sn-glycerol 3-phosphoric acid	Organic acid	Negative	P containing
	Uric acid	Organic acid	Negative	N containing
	Hexose phosphoric acid	Other	Negative	P containing
	Pterin	Other	Positive	N containing
	Xanthine	Other	Negative	N containing
Mix, *Agrobacterium, Pseudomonas*	Alanine	Amino acid	Negative	N containing
	Choline	B vitamin	Positive	N containing
Mix, *Arthrobacter*	Dihexose 1	Carbohydrate	Negative	No
Mix, *Arthrobacter, Agrobacterium*	N-Acetyl-Glutamic acid	Amino acid derivative	Negative	N containing
	Dihexose 2	Carbohydrate	Positive	No
	Adenosine	Nucleoside	Positive	N containing
	Guanosine	Nucleoside	Negative	N containing
	Inosine	Nucleoside	Negative	N containing
	Uridine	Nucleoside	Negative	N containing
	Cytosine	Nucleotide	Positive	N containing
	Guanine	Nucleotide	Positive	N containing
	3-(4-hydroxyphenyl)lactic acid	Organic acid	Negative	No
Mix, *Arthrobacter, Pseudomonas*	Nicotinamide	B vitamin	Positive	N containing
	Adenine	Nucleobase	Negative	N containing
	Uridine-5-monophosphoric acid	Nucleotide derivative	Negative	Both N and P containing
*Pseudomonas*	N-Acetyl-Lysine	Amino acid derivative	Positive	N containing
	Gluconic acid	Organic acid	Negative	No

Several of these necromass-derived metabolites decreased over the 14-day incubation, with different temporal patterns of metabolite depletion across the different necromass treatments. By Day 2, more metabolites were depleted (negative log-fold change from time-0, *P* < .05) in the mixed necromass treatment, compared to the individual necromass treatment (Fig. 2). For instance, guanosine, inosine, and gluconic acid were depleted only by Day 4 for the individual treatment but were depleted by Day 2 within the mixed necromass. The majority of identified amino acids, nucleic acid components, and organic acids were significantly depleted by the end of the incubation from *Agrobacterium* necromass. The mixed- and *Pseudomonas*-necromass followed this trend, albeit to lesser extents. Eight metabolites were significantly depleted only from *Agrobacterium* necromass, despite being present in other necromass. Across all time points, *Arthrobacter* necromass appeared to have the fewest depleted metabolites relative to the other two necromass strains. Two phosphate-containing isomers: glycerol 2-phosphoric acid/sn-glycerol 3-phosphoric acid and a hexose phosphoric acid were universally depleted across all treatments (Fig. 2).

### Community composition of necromass-utilizers

**Figure 3:**
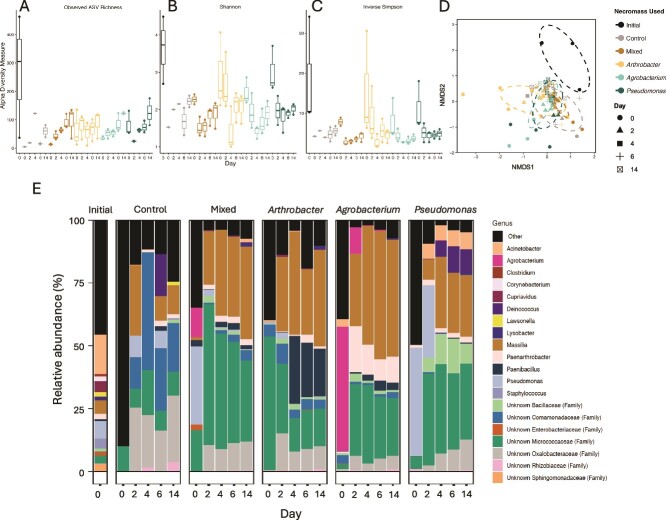
Alpha and beta diversity of 16S communities across necromass treatment and day of collection. Alpha diversity (rarefied) of (A) observed ASVs, (B) Shannon diversity, and (C) inverse Simpson for each necromass strain over the 14-day experiment. The horizontal line is the diversity metric of the initial inoculum from the sediment microbiome. (D) NMDS analysis of the ASVs of the bacterial communities under different necromass over time. For A–D, different necromass treatments are depicted by different colors. (E) Stacked bar plot of relative abundance of top genera across the necromass treatments and time (>0.01% of total 16S copies). The ASVs for each necromass strain were removed from these relative abundance data.

We did not find a significant effect of necromass type on microbial community alpha diversity, but did find support for the effect of incubation time. There was a significant increase in richness (number of unique ASVs observed) by Day 14 compared to Days 0 and 2 (Wilcox, *P* = .007; BH adjusted; Fig. 3A). There was a slight decrease in Shannon diversity by the second day (Wilcox, *P* = .06, BH adjusted; Fig. 3B), but was significantly greater by Day 14 compared to Day 2 (*P* = .03). It is important to note that our findings may be affected by the low read recovery observed for earlier timepoints of non-necromass ASVs, as up to 99% of the initial T0 and Day-2 sequence samples comprised necromass ASV reads (Supplemental Figs. S1 and S2). Archaea were detected in the initial inoculum (3.6 ± 2.6% relative abundance) but became significantly less abundant throughout the incubation across all treatments. Therefore, we focus the rest of our analysis on bacterial taxa.

PERMANOVA analysis revealed significant effects of both necromass (Fig. 3D, F_4,56,_  *P* = .001, R^2^ = 0.18) and sampling time point (*P* = .001, R^2^ = 0.19) on community composition. Additionally, community structure was affected by whether necromass added was derived from a single isolate or from multiple isolates, although to a lesser extent compared to time (PERMANOVA, necromass R^2^ = 0.04, *P* = .01; timepoint R^2^ = 0.19, *P* = .001).

The most abundant taxa in this study responded in a similar fashion to all necromass treatments, regardless of the necromass source. Oxalobacteraceae, particularly *Massilia* and to a lesser extent *Noviherbaspirillum*, initially accounted for a small portion of the community (0.15%–3%). However, their relative abundance increased significantly within the first 2 days of incubation (13%–53%) and reached 36%–52% by the end of the incubation across all treatments, including the no-necromass control (Fig. 3E; Supplemental Fig. S3; Supplemental Fig. S4). While *Massilia* exhibited substantial relative growth across treatments, necromass addition resulted in greater absolute growth compared to the no-necromass control (ANCOM-BC *P* < .05; Supplemental Fig. S3; Supplemental Fig. S4). Similarly, Micrococcaceae, including *Paenarthrobacter*, maintained high relative abundance across all treatments and time points (particularly in the *Agrobacterium* necromass), comprising 15%–34% of the community by Day 14 (Fig. 3E; Supplemental Fig. S3; Supplemental Fig. S4).

### Inferred functional abundance of necromass-metabolite utilizing microbial taxa

**Figure 4:**
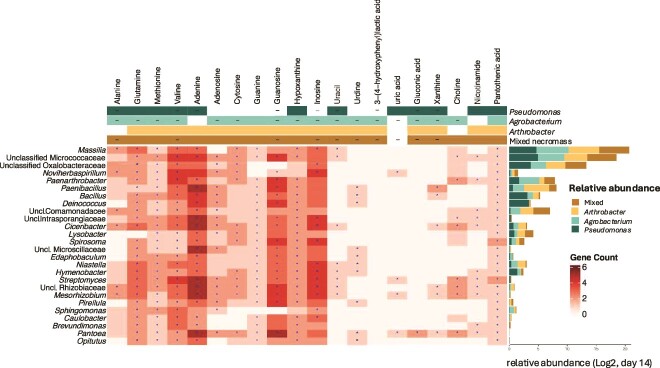
Heatmap showing gene counts of metabolic pathways for genera responding to amended necromass metabolites, as imputed from the PICRUSt2 pipeline. Metabolic groups corresponding to each metabolite are annotated at the top of the heatmap. The log2 scale of the average relative abundance of these genera is shown as bar plot on the right of the heatmap. The numbers of genes indicate the cumulative total of EC pathway genes corresponding to each metabolite. Pathways marked with an asterisk (*) have at least one complete degradation pathway that is fully identified according to MetaCyc and confirmed by PICRUSt2 results.

We leveraged PICRUSt2 to provide functional insights and to complement the taxonomic information obtained from 16S rRNA gene sequencing and metabolite analysis. We explored if there were links between specific taxa to potential utilization of necromass-derived metabolites, and whether genera significantly enriched by the end of the experiments would also possess degradation pathways for utilizing the metabolites depleted. From the metabolomics data, alanine, valine, guanosine, inosine, uracil, 3-(4-hydroxyphenyl) lactic acid, gluconic acid, and xanthine were significantly depleted in three out of four necromass treatments (Figs. 2,4). However, PICRUSt2 analysis revealed that only four metabolites (glutamine, methionine, valine, and pantothenic acid) could be universally utilized by all responding genera and had complete degradation pathways. One possible explanation is that the dominant responding genera, due to their complete degradation pathways, are primarily responsible for driving the depletion of these key metabolites. For example, the most abundant genus, *Massilia*, possesses predicted pathways to fully metabolize alanine, inosine, and uracil. Similarly, *Noviherbaspirillum* was one of four genera capable of completely degrading uric acid. Also, xanthine utilization may be attributed to *Paenibacillus* and *Bacillus*, both of which constitute a substantial proportion of the overall community. However, some discrepancies between the LC/MS and PICRUSt2 results remain unexplained. For instance, LC/MS detected gluconic acid and 3-(4-hydroxyphenyl)lactic acid, but the pathways for their degradation are not fully accounted for. Very few responding ASVs showed a complete pathway for their utilization, with only *Pantoea* for gluconic acid and none for 3-(4-hydroxyphenyl)lactic acid. Conversely, PICRUSt2 results indicated that most or all of the responding genera possess complete degradation pathways for guanine and pantothenic acid, while LC/MS measurements revealed no significant utilization, revealing some limitations of PICRUSt2 analysis.

## Discussion

Here we investigated the impact of microbial necromass on a dynamic subsurface microbial community, focusing on hydrophilic soluble metabolites. The change in community composition and necromass metabolite profile followed similar temporal response patterns as demonstrated previously [13], showing an initial lag in response, followed by rapid decrease in necromass compounds, supported by over a 100-fold increase in bacterial cells by 2 weeks (Fig. 1A). The enriched communities were also less diverse during these periods of rapid metabolite consumption compared to the initial inoculum but eventually became more diverse over time (Fig. 3A–C), indicating that initial responders to necromass may be less diverse and more copiotrophic [30]. This pattern highlights the dynamic nature of microbial community growth in response to metabolites in necromass over time. However, due to the low read-recovery of ASVs that were not derived from the necromass strains early in the experiment, caution should be exercised when interpreting temporal changes to community composition, and future studies with improved read recovery are warranted to further validate the collective observed trends.

Our results demonstrate that necromass metabolites from some microbial taxa may be more recalcitrant to bacterial utilization than others. The gram-positive *Arthrobacter* necromass generally appeared less preferred compared to gram-negative *Agrobacterium* and *Pseudomonas* necromass over time, despite numerous metabolites in comparable concentrations across the three necromass strains. This has been observed before, Dong *et al*. [25] traced necromass using H_2_^18^O stable isotope probing, and found different decomposition patterns for multiple taxa that contributed to necromass, and that *Arthrobacter* necromass appeared relatively recalcitrant.

Microbial necromass from different bacterial species resulted in variations in community composition and the enrichment of specific microbes. However, the individual necromass and mixed-necromass generally elicited similar responses in terms of the compounds utilized and the bacterial taxa that responded. Notably, *Massilia* may drive uptake of the commonly-depleted necromass metabolites of alanine, inosine, and uracil, as it is known to possess the complete utilization pathways for these compounds. *Massilia* has been identified as a notable responder to necromass in other ecosystems as well, both in surface soil [25], as well as groundwater [13]. Micrococcaceae in surface soils has similarly been found to be notable utilizers of microbial residues, growing rapidly on extracellular microbial DNA [26] and amino acids [44]. These results suggest that specific bacterial taxa may consistently be important initial responders to microbial necromass across different ecosystems. However, while individual bacterial taxa may drive some facets of necromass metabolism and recycling, considering the broader context of whole-community interactions is also essential. The observed discrepancies between the metabolomics and PICRUSt2 results suggest that a large fraction of necromass catabolic pathways are not wholly driven by individual taxa. For instance, when grown in isolation, *Massilia* was unable to grow on the same necromass added to the entire community (Supplemental Fig. S6), indicating the potential need for cross-feeding with other taxa. Similarly, *Spirosoma*, which also positively responded across necromass types, displayed limited necromass-growth in isolation. However, while *Deinococcus* predominately responded only to *Pseudomonas* necromass, it grew readily on both *Arthrobacter* and *Pseudomonas* necromass when grown in isolation, suggesting interaction-inhibited growth. Therefore, the collective metabolic activities and interactions within the entire microbial community may be more crucial in driving overall necromass utilization and recycling. To better link microbial functions with metabolite utilization in these systems, additional evidence from transcriptomic analyses would be required.

In terms of more consistently transient necromass metabolites, bioavailable phosphate-containing compounds will quickly be incorporated into living biomass. We also observed the rapid depletion of phosphate-containing metabolites across necromass types. In subsurface environments, phosphate is poorly available, so microbes generally mine for phosphates via release of organic acids or expression of phosphatase enzymes [45]. Microbial residues are indirectly regulated by stoichiometric constraints, such as C:N:P ratios [8]. Microorganisms typically adjust their carbon use efficiency to maintain a stoichiometric balance between the available substrate and their nutrient demands [46]. Overall, this study demonstrates that necromass could serve as a source of readily available phosphorus, particularly in nutrient-poor systems.

While we detected specific metabolites bioavailable to the microbial community, we also found specific metabolites that may be more persistent in subsurface systems. Of the identified metabolites, there was a greater variation in initial metabolite composition across the different necromass treatments, but metabolic composition became more similar over time (Supplemental Fig. S5). This suggests that while different metabolites were depleted over time, the more persistent metabolites were similar across the different necromass. For instance, compounds that should be highly bioavailable, such as guanine, alanine, and adenine, did not consistently decrease. This indicates either comparable turnover of those metabolites (with no net change), or resistance to microbial utilization. From a recent study investigating the persistence of various components of soil microbial necromass using ^13^C tracers, metabolites were both a large fraction of microbial necromass C, as well as an order of magnitude more enriched in ^13^C than any other necromass pool, despite contributing to only 0.3% of the total soil C pool [31].

Due to the inoculum unavoidably containing trace amount of necromass (as cell growth is not synchronized and even mid-log phase growth will contain some dead cells), we acknowledge the presence of necromass in the control treatment. However, the TOC of the inoculum was <1 ppm carbon, and the total number of cells in the inoculum was 1 × 10^6^ cells ml^−1^ (with 3.5 × 10^5^ live cells ml^−1^), we can consider the control as a necromass treatment, albeit with >30 times less necromass carbon. This was reflected in both the low microbial growth compared to the added-necromass treatments (Fig. 1A), as well as similar convergence of both community and metabolite composition by the end of the experiment (Fig. 3D, Supplemental Fig. S5). This supports that regardless of necromass type, there may be predictable patterns in necromass-community responses as well as what necromass-derived metabolites are relatively stabilized in belowground systems [10, 31].

Studies investigating necromass utilization have attempted to quantify specific compounds, such as extracellular DNA, as proxies for the larger necromass pool [26, 47]. We observed a decrease in ASV copy number and multiple nucleic acid constituents (e.g. adenine, uracil, adenosine, and guanosine), as well as enriched purine and pyrimidine metabolic pathways (Supplemental Tables S4, S5), supporting the importance of extracellular nucleic acids in necromass recycling [26, 47]. We used changes in necromass-derived ASV relative abundance to proxy necromass catabolism over time and microbial growth from necromass. Metabolomic responses also indicated bacterial utilization of added necromass, particularly nucleic acid constituents. For instance, uracil was abundant in all necromass lysates and generally depleted during incubation, highlighting this RNA nucleobase’s accessibility for microbial processing. Notably, adenine was significantly depleted only from *Pseudomonas* necromass, while thymine decreased for all treatments (including the more-persistent *Arthrobacter* necromass) except *Pseudomonas*. This suggests both uptake and recycling of extracellular nucleic acid fragments for growth [26, 48] and non-growth-related microbial activity [49]. These results, consistent with other studies, indicate necromass-derived nucleic acids were incorporated into living biomass, adsorbed to sediment minerals, or fragmented beyond sequencing capability. Differences in utilization between purines and pyrimidines highlight the dynamic nature of nucleic acid decomposition, warranting further investigation.

Like extracellular DNA, amino sugar biomarker quantification such as muramic acid and glucosamine [50, 51] has also been used to estimate microbial necromass contribution to NOM formation [23, 50] but only accounts for cell wall components. If still bound to cell wall fragments, these amino sugars would have been filtered out of the lysate supernatant used as a substrate in the incubation. A constraint of this study is that we focused solely on the fate of low molecular weight hydrophilic metabolites (<1050 *m/z*), which represent only a fraction (9%–25%) of the total necromass material [52]. Our study demonstrates a nuanced response of some of the diverse low-molecular weight compounds from necromass.

Microbial necromass is frequently treated as a single, homogenous carbon pool. This study characterizes a poorly-studied component of microbial necromass, metabolites, as an important, heterogenous, and dynamic contributor to OM [53]. Subsurface ecosystems typically are limited in organic carbon, especially regular fresh inputs of photosynthetic-derived OM [1]. Recycling of organic-carbon present in subsurface environments is critical for the microorganisms that reside there. From this study, we found further support that microbial necromass is a critical carbon source for subsurface microorganisms, and that diverse subsurface bacteria can use necromass as a C source [5]. Although there was some variation, abundant taxa quickly utilized several components of necromass C, regardless of the strain used to generate the necromass, and some components persisted as subsurface OM. Deeper investigation of interactions between different minerals and specific necromass compounds [31,54,55], with different microbial communities, could advance our understanding of microbial-mediated cycling of more persistent necromass components. Furthermore, incorporating additional ‘omics techniques (metagenomic, metatranscriptomic, and/or proteomic) would allow for greater understanding of validated metabolic pathways involved in necromass utilization, as well as a more complete understanding of transformation of high molecular weight fractions of necromass such as cell wall components and proteins. Lastly, coupling methods to differentiate between living and dead microbial biomass, as well as deeply characterizing the necromass pool and subsequent microbial transformations, may improve our understanding of subsurface necromass recycling and persistence.

## Supplementary Material

Necromass_Supplemental_Materials_final_ycaf006

Table_S1_LCMS_parameters_ycaf006

Table_S2_Identified_Metabolites_ycaf006

Table_S3_pH_ycaf006

Table_S4_metabolites_predicted_pathways_ycaf006

Table_S5_KO_predicted_pathways_ycaf006

## Data Availability

Raw 16S sequence data from this study are available in the NCBI short read archive under accession PRJNA1137404. The raw MS data are available as a MassIVE dataset at https://massive.ucsd.edu/ (ID:MSV000095159).
