# Supplementary material for: Unraveling the influence of microbial necromass on subsurface microbiomes: metabolite utilization and community dynamics
Source: ISME Commun. 2025 Jan 29;5(1):ycaf006. doi: 10.1093/ismeco/ycaf006 (PMC11843093; doi:10.1093/ismeco/ycaf006)
Supplement: Necromass_Supplemental_Materials_final_ycaf006 [file necromass_supplemental_materials_final_ycaf006.pdf]

Unraveling the Influence of Microbial Necromass on Subsurface Microbiomes: Metabolite Utilization and Community Dynamics

Brianna K. Finley[1], Brandon C. Enalls[1], Markus de Raad[2], Mariam Al Said[1], Mingfei Chen[1], Dominique C. Joyner[3], Terry C. Hazen[3,4], Trent R. Northen[2,5], Romy Chakraborty[1]

[1]Department of Ecology, Earth and Environmental Sciences Area, Lawrence Berkeley National Laboratory, Berkeley, CA, United States,

[2]Environmental Genomics and Systems Biology Division, Lawrence Berkeley National Laboratory, Berkeley, CA, United States

[3]University of Tennessee, Knoxville, Tennessee, USA

[4]Oak Ridge National Laboratory, Oak Ridge, Tennessee, USA

[5]Joint Genome Institute, Lawrence Berkeley National Laboratory, Berkeley, CA, United States

Supplementary Methods

Growth curves of potential necromass-utilizing isolates

Based on ASVs of bacteria whose abundance increased due to necromass addition, we identified strains within our extensive isolate collection with matching ASVs. These isolates belonged to *Massilia, Deinococcus, Paenarthrobacter, Pantoea, Pedobacter,* and *Spirosoma* spp. Isolates were reconstituted from glycerol stocks on Reasoner's 2A agar (R2A) plates at 30oC. Single colonies were then grown in 5mL R2A liquid media at 30°C, shaken at 150rpm until OD reached ~0.3. Cultures were then centrifuged at 6500g for 10 minutes, supernatant removed, then culture pellets were washed in 1 mL phosphate buffered saline three times. Each isolate was inoculated in clear 96 well plates at 10% volume in 200 uL total volume of synthetic groundwater medium with either 1X and 0.5X necromass of the *Pseudomonas* necromass lysate as used in the enrichment incubation, or 1X and 0.5X *Arthrobacter* necromass lysate. We did not have sufficient *Agrobacterium* necromass lysate remaining from the main experiment for these isolate growth curves. For the *Pseudomonas* necromass, the concentrations were 134 ppm C for 1X and 67 ppm C for 0.5X. For the *Arthrobacter* necromass, the concentrations were 96 ppm C for 1X and 48 ppm C for 0.5X. Optical density at 600 nm (OD600) was measured on Epoch2 microplate readers (BioTek) every hour for 72 hours, and adjusted based on negative controls (without the isolate).

Supplementary Figures

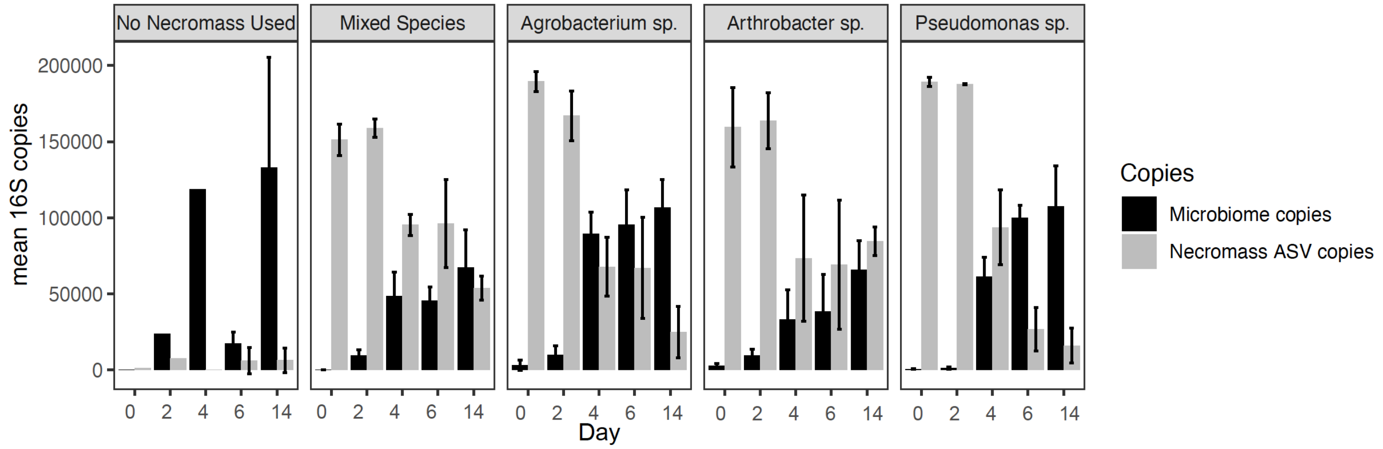

Figure S1. Sequencing depth (16S copies recovered) between that of the identified necromass ASVs of the *Agrobacterium*, *Arthrobacter* and *Pseudomonas* strains and the enriched bacterial microbiome over time.

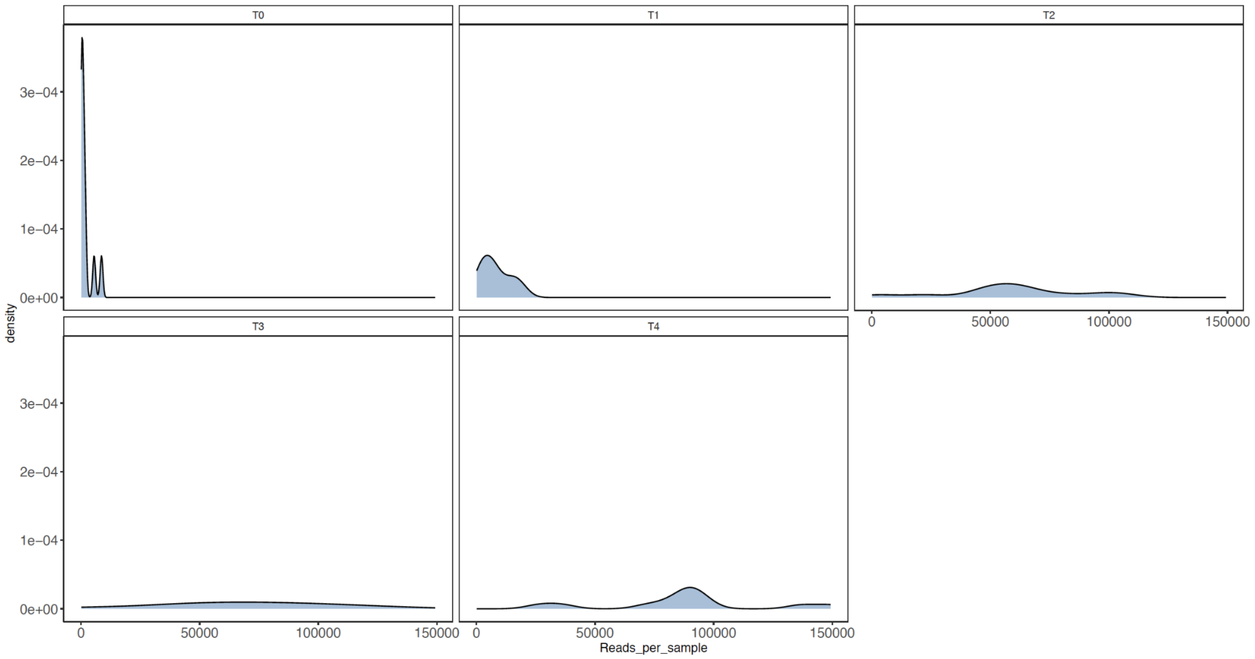

Figure S2. Read distribution of non-necromass asvs (microbial community) over time.

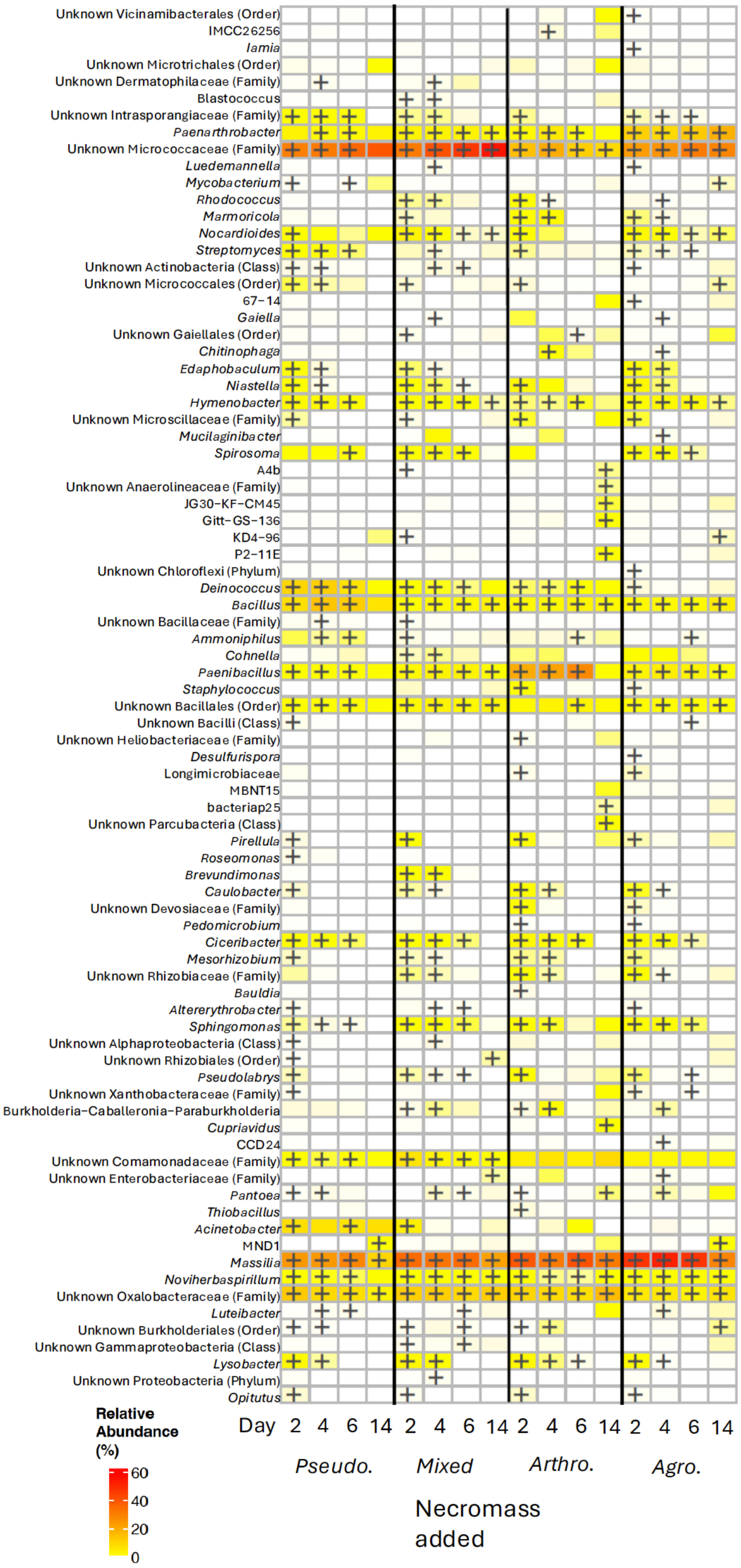

Unknown Vicinamibacterales (Order)
IMCC26256
Iamia
Unknown Microtrichales (Order)
Unknown Dermatophilaceae (Family)
Blastococcus
Unknown Intrasporangiaceae (Family)
Paenarthrobacter
Unknown Micrococcaceae (Family)
Luedemannella
Mycobacterium
Rhodococcus
Marmoricola
Nocardioides
Streptomyces
Unknown Actinobacteria (Class)
Unknown Micrococcales (Order)
67−14
Gaiella
Unknown Gaiellales (Order)
Chitinophaga
Edaphobaculum
Niastella
Hymenobacter
Unknown Microscillaceae (Family)
Mucilaginibacter
Spirosoma
A4b
Unknown Anaerolineaceae (Family)
JG30−KF−CM45
Gitt−GS−136
KD4−96
P2−11E
Unknown Chloroflexi (Phylum)
Deinococcus
Bacillus
Unknown Bacillaceae (Family)
Ammoniphilus
Cohnella
Paenibacillus
Staphylococcus
Unknown Bacillales (Order)
Unknown Bacilli (Class)
Unknown Heliobacteriaceae (Family)
Desulfurispora
Longimicrobiaceae
MBNT15
bacteriap25
Unknown Parcubacteria (Class)
Pirellula
Roseomonas
Brevundimonas
Caulobacter
Unknown Devosiaceae (Family)
Pedomicrobium
Ciceribacter
Mesorhizobium
Unknown Rhizobiaceae (Family)
Bauldia
Altererythrobacter
Sphingomonas
Unknown Alphaproteobacteria (Class)
Unknown Rhizobiales (Order)
Pseudolabrys
Unknown Xanthobacteraceae (Family)
Burkholderia−Caballeronia−Paraburkholderia
Cupriavidus
CCD24
Unknown Comamonadaceae (Family)
Unknown Enterobacteriaceae (Family)
Pantoea
Thiobacillus
Acinetobacter
MND1
Massilia
Noviherbaspirillum
Unknown Oxalobacteraceae (Family)
Luteibacter
Unknown Burkholderiales (Order)
Unknown Gammaproteobacteria (Class)
Lysobacter
Unknown Proteobacteria (Phylum)
Opitutus
Relative Abundance (%)
60
40
20
0
Day 2 4 6 14 2 4 6 14 2 4 6 14 2 4 6 14
Pseudo.
Mixed
Arthro.
Agro.
Necromass added

Figure S3. Heatmap of relative abundance (percent of community within treatment) of globally significant genera from the no-necromass control across all incubation time points from Analysis of Compositions of Microbiomes with Bias Correction (ANCOM-BC).

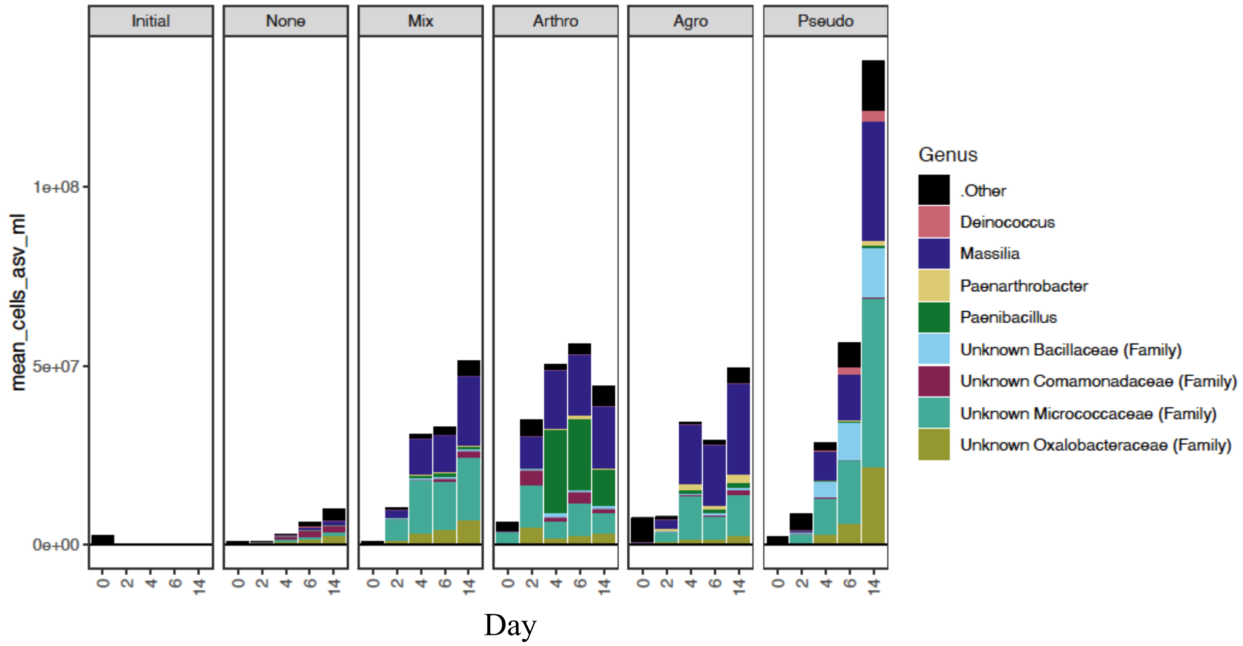

Figure S4. Estimated absolute growth of top responding genera as mean cells mL-1. Each asv within each genus was normalized by predicted 16S copy number via PICRUSt2 and by the total number of cells grown over time as determined by flow cytometry.

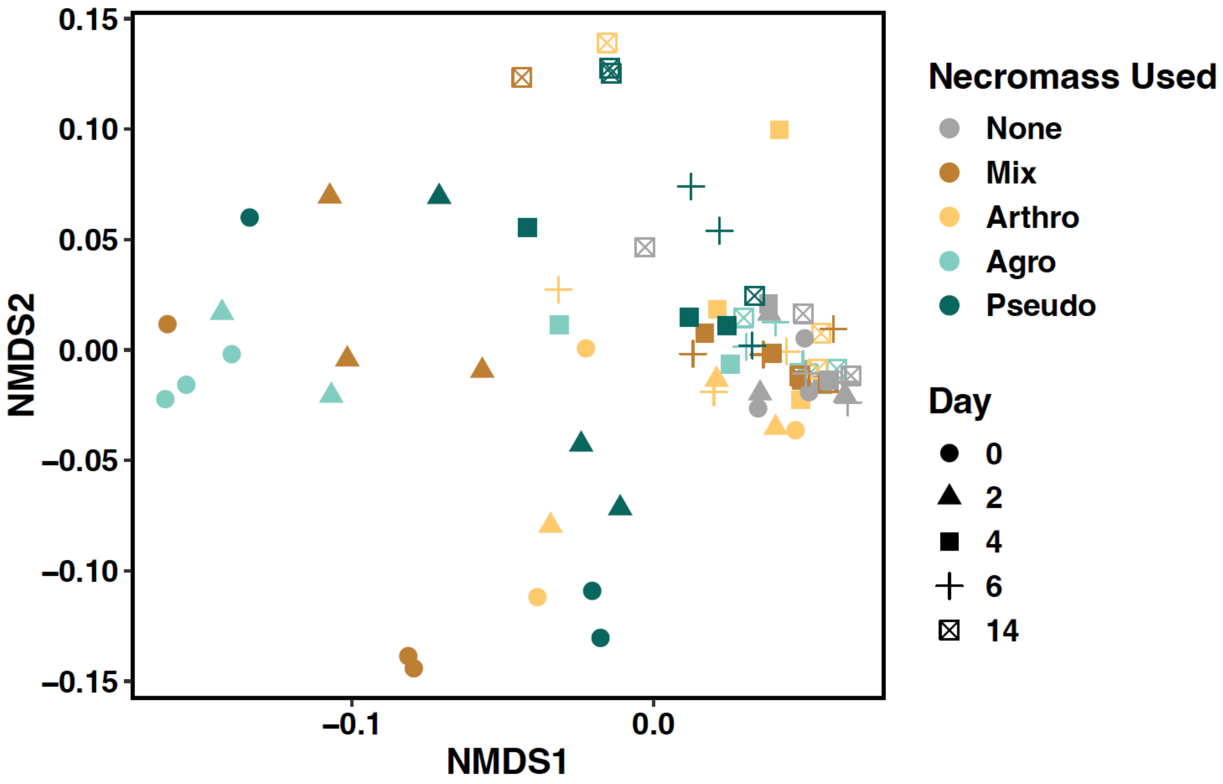

Figure S5. NMDS of annotated metabolites (Bray-Curtis dissimilarity matrix). Different colors indicate the necromass treatment, and symbols indicate the sampling day.

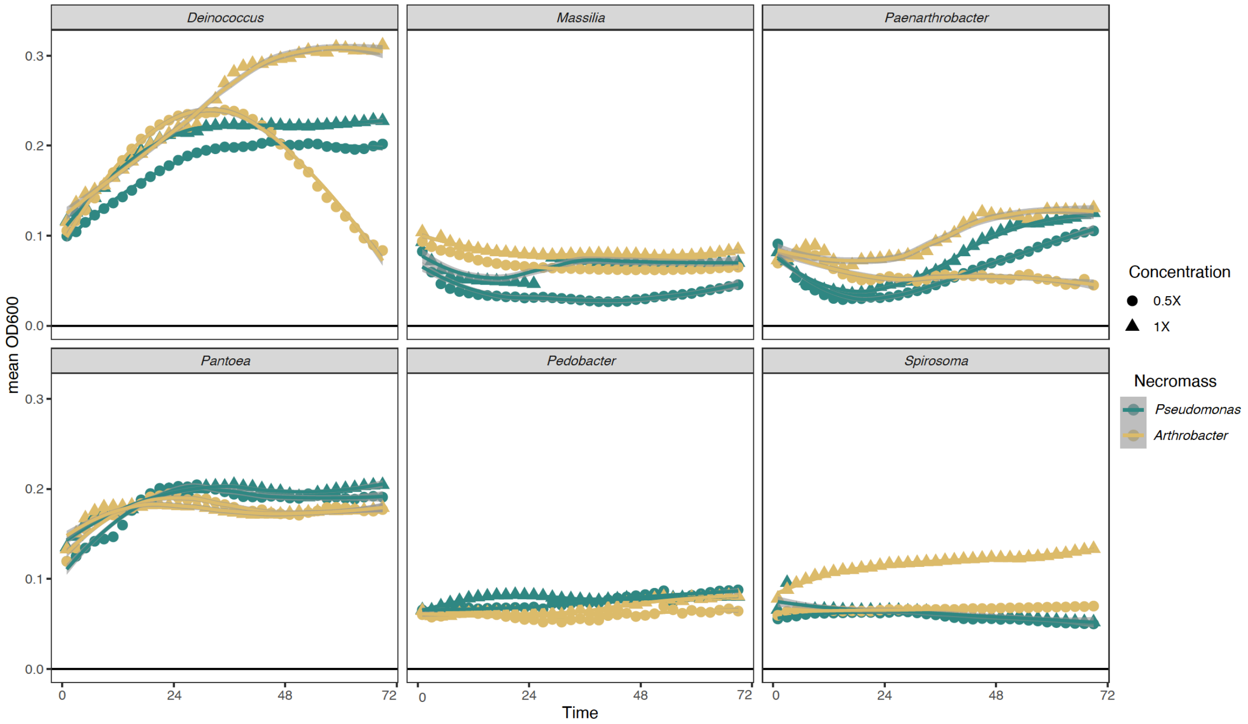

Figure S6. Growth curves (mean OD600) of isolates matching the ASVs of taxa that positively responded to added necromass (*Pseudomonas* or *Arthrobacter*) within the community as identified by differential abundance analysis over 72 hours. 1x concentration is 96 ppm C for *Arthrobacter* and 134 ppm C for *Pseudomonas*, with 0.5x concentration half, respectively.
