# Supplementary material for: Unraveling the influence of microbial necromass on subsurface microbiomes: metabolite utilization and community dynamics
Source: ISME Commun. 2025 Jan 29;5(1):ycaf006. doi: 10.1093/ismeco/ycaf006 (PMC11843093; doi:10.1093/ismeco/ycaf006)
Supplement: Table_S3_pH_ycaf006 [file table_s3_ph_ycaf006.docx]

Table S3. Mean pH (± standard error of the mean, n=3) of the synthetic groundwater-sediment slurry under various necromass additions over the 14-day incubation.

| Treatment | T0 | day 2 | day 4 | day 6 | day 14 |
| --- | --- | --- | --- | --- | --- |
| No Necromass | 6.23 ± 0.09 | 6.3 ± 0.10 | 6.3± 0.09 | 6.36± 0.09 | 6.52±0.05 |
| Mixed species | 6.46±0.03 | 6.48±0.04 | 6.54±0.03 | 6.58±0.003 | 6.75±0.01 |
| Agrobacterium sp. | 6.30±0.02 | 5.92±0.42 | 6.45±0.01 | 6.47±0 | 6.67±0.02 |
| Pseudomonas sp. | 6.76±0.11 | 6.80±0.08 | 6.75±0.05 | 6.87±0.07 | 6.94±0.06 |
| Arthrobacter sp. | 6.53±0.08 | 6.44±0.06 | 6.55±0.04 | 6.60±0.04 | 6.82±0.02 |
