# Supplementary material for: Unraveling the influence of microbial necromass on subsurface microbiomes: metabolite utilization and community dynamics
Source: ISME Commun. 2025 Jan 29;5(1):ycaf006. doi: 10.1093/ismeco/ycaf006 (PMC11843093; doi:10.1093/ismeco/ycaf006)
Supplement: Table_S4_metabolites_predicted_pathways_ycaf006 [file table_s4_metabolites_predicted_pathways_ycaf006.docx]

Table S4: Result of metabolites identified as necromass-derived on potential pathways. The total is the total number of compounds in the pathway, and hits are the matched compounds (metabolites) derived from necromass. P-values are presented as raw, -log10, adjusted by the Holm Bonferroni method, and adjusted using the false discovery rate (FDR). Impact refers to the pathway impact calculated from pathway topology analysis.

|  | Total | Expected | Hits | Raw p | -logl0(p) | Holm adjust | FDR | Impact |
| --- | --- | --- | --- | --- | --- | --- | --- | --- |
| Purine metabolism | 53 | l.56 | 7 | 4.02.E-04 | 3.40.E+00 | 2.8l.E-02 | 2.8l.E-02 | 0.04 |
| Pyrimidine metabolism | 34 | l.00 | 5 | 2.07.E-03 | 2.68.E+00 | l.43.E-0l | 7.26.E-02 | 0.27 |
| Arginine biosynthesis | l3 | 0.38 | 2 | 5.30.E-02 | l.28.E+00 | l.00.E+00 | l.00.E+00 | 0.24 |
| Carbapenem biosynthesis | 3 | 0.09 | l | 8.6l.E-02 | l.07.E+00 | l.00.E+00 | l.00.E+00 | 0.00 |
| beta-Alanine metabolism | 3 | 0.09 | l | 8.6l.E-02 | l.07.E+00 | l.00.E+00 | l.00.E+00 | 0.00 |
| Nitrogen metabolism | 4 | 0.l2 | l | l.l3.E-0l | 9.46.E-0l | l.00.E+00 | l.00.E+00 | 0.00 |
| Biosynthesis of various plant secondary | 4 | 0.l2 | l | l.l3.E-0l | 9.46.E-0l | l.00.E+00 | l.00.E+00 | 0.00 |
| metabolites |  |  |  |  |  |  |  |  |
| Glycerophospholipid metabolism | l2 | 0.35 | l | 3.04.E-0l | 5.l7.E-0l | l.00.E+00 | l.00.E+00 | 0.00 |
| Glyoxylate and dicarboxylate | l3 | 0.38 | l | 3.25.E-0l | 4.88.E-0l | l.00.E+00 | l.00.E+00 | 0.00 |
| metabolism |  |  |  |  |  |  |  |  |
| Nicotinate and nicotinamide metabolism | l3 | 0.38 | l | 3.25.E-0l | 4.88.E-0l | l.00.E+00 | l.00.E+00 | 0.04 |
| Alanine, aspartate and glutamate | l3 | 0.38 | l | 3.25.E-0l | 4.88.E-0l | l.00.E+00 | l.00.E+00 | 0.l9 |
| metabolism |  |  |  |  |  |  |  |  |
| Histidine metabolism | l4 | 0.4l | l | 3.45.E-0l | 4.62.E-0l | l.00.E+00 | l.00.E+00 | 0.00 |
| -Amin0 acid metabolism | l4 | 0.4l | l | 3.45.E-0l | 4.62.E-0l | l.00.E+00 | l.00.E+00 | 0.00 |
| Porphyrin metabolism | l5 | 0.44 | l | 3.65.E-0l | 4.38.E-0l | l.00.E+00 | l.00.E+00 | 0.00 |
| Arginine and proline metabolism | l6 | 0.47 | l | 3.84.E-0l | 4.l5.E-0l | l.00.E+00 | l.00.E+00 | 0.00 |
| Pantothenate and CoA biosynthesis | 2l | 0.62 | l | 4.72.E-0l | 3.26.E-0l | l.00.E+00 | l.00.E+00 | 0.l2 |
| Pentose phosphate pathway | 24 | 0.7l | l | 5.l9.E-0l | 2.85.E-0l | l.00.E+00 | l.00.E+00 | 0.00 |
| Cysteine and methionine metabolism | 38 | l.l2 | l | 6.90.E-0l | l.6l.E-0l | l.00.E+00 | l.00.E+00 | 0.l5 |
